# Targeting Ferroptosis to Eliminate Senescent Cells: Mechanisms and Therapeutic Potential

**DOI:** 10.14336/AD.2025.0141

**Published:** 2026-06-30

**Authors:** Sanjay Kumar Kureel, Blake B. Rasmussen

**Affiliations:** ^1^Barshop Institute for Longevity & Aging Studies, The University of Texas Health Science Center at San Antonio, San Antonio, Texas, USA.; ^2^Department of Cellular & Integrative Physiology, The University of Texas Health Science Center at San Antonio, San Antonio, Texas, USA.

**Keywords:** Senescence, Anti-apoptotic, Senolytics, Ferroptosis, Lipid peroxidation, Oxidative damage

## Abstract

Cellular senescence is involved in early development, wound healing, and tumor suppression. However, the accumulation of senescent cells (SCs) drives tissue dysfunction and many age associated pathologies such as cancer and neurodegeneration. SCs demonstrate irreversible cessation of cell cycle, overexpression of anti-apodotic proteins, and senescence associated secretory phenotype (SASP), cause tissue dysfunction. Traditional senolytics induces apoptosis but have poor selectivity, uncertain long-term efficacy, and resistant SCs, limiting their use. Ferroptosis, an iron-dependent, non-apoptotic form of programmed cell death, has emerged as a promising alternative. SCs bypass the apoptosis by overexpression of an anti-apoptotic pathway, but ferroptosis uses oxidative damage to overcome these defenses, thus, making it effective for eliminating SCs. This review critically evaluates ferroptosis-mediated processes such as elevated level of iron, polyunsaturated fatty acids (PUFAs) and oxidative damages in elimination of SCs and its therapeutic potential for age related pathologies including fibrosis, cancer and neurodegenerative diseases. This review highlights the molecular mechanisms underlying ferroptosis and its potential for treating age-related diseases such as fibrosis, atherosclerosis, osteoarthritis, and neurodegeneration. By addressing the translational challenges of ferroptosis-based therapies, we emphasize its potential as a next generation senolytic for targeting senescence and aging-related pathologies.

## 1. Introduction:

Aging is characterized by a progressive and irreversible decline in biological functions, with profound implications for social structures and increased susceptibility to a spectrum of diseases [1-3] Among the thirteen hallmarks of aging delineated by Lopez group [4, 5], the accumulation of SCs stands out as a pivotal contributor to age-related deterioration [6]. SCs are defined by features such as irreversible cell cycle arrest, resistance to apoptosis, and the secretion of pro-inflammatory cytokines, chemokines, proteases, collectively termed the SASP [7]. These characteristics significantly exacerbate aging and the onset of age-related disorders [8].

Conventional approaches to counteract the deleterious effects of SCs, notably senolytic therapies, aim to induce apoptosis in these cells [9-11]. However, these strategies are hindered by limitations, including incomplete clearance of SCs and off-target toxicities, highlighting the need for innovative alternatives [12, 13]. Ferroptosis, an iron-dependent form of programmed cell death distinct from apoptosis, necrosis, and autophagy, emerges as a promising candidate. Characterized by the accumulation of lipid peroxides in an iron-dependent manner, ferroptosis offers a unique mechanism for targeting senescent cells [14, 15]. The heightened susceptibility of senescent cells to ferroptosis stems from their altered iron metabolism and elevated levels of reactive oxygen species (ROS)[16, 17], with SASP factors potentially amplifying this vulnerability [18, 19].

This review examines the role of ferroptosis in the selective elimination of senescent cells, with a particular emphasis on the molecular mechanisms underlying their susceptibility to this form of cell death [20]. It further evaluates the comparative advantages of ferroptosis-based therapies over traditional senolytic approaches and addresses the therapeutic potential [21] and challenges associated with their translation into clinical applications.

## 2. Overview of Senescence:

Cellular senescence, characterized by irreversible cell cycle arrest, is a fundamental process that supports tissue development and serves as a safeguard against malignant transformation [8]et al., 2018). However, with advancing age, the accumulation of SCs due to impaired immune clearance disrupts tissue homeostasis and promotes chronic inflammation through the SASP, contributing to age-related diseases such as cancer and osteoarthritis [22, 23]. Senescent cells exhibit hallmark features, including permanent withdrawal from the cell cycle, secretion of pro-inflammatory molecules via SASP, suppression of pro-apoptotic proteins, and metabolic reprogramming [8, 24]. Additionally, these cells undergo biophysical alterations, such as enlarged morphology and reduced motility [25, 26]. Although common markers, such as p53 overexpression and elevated reactive oxygen species (ROS) levels, are associated with senescence, the heterogeneity across tissues precludes the identification of a universal marker [7, 27].

During development, SCs contribute to tissue morphogenesis and repair, with their clearance tightly regulated by immune cells, including macrophages and natural killer (NK) cells. However, immune aging disrupts this regulatory process, leading to the accumulation of senescent cells [28, 29]. SASP exacerbates inflammation, fostering chronic conditions such as osteoporosis, cancer, and fibrosis [23, 27].

Senescence plays a complex, dual role in cancer, acting as both a tumor suppressor and a promoter of tumor progression. In early tumorigenesis, senescence serves as a protective mechanism by halting uncontrolled proliferation, triggered by oncogene activation (RAS) or DNA damage, and mediated through the p53/p21 or p16/Rb pathways (Collado [30] & Serrano, 2010). This tumor-suppressive function is evident in premalignant lesions, where SCs arrest cancer development. Conversely, in established cancers, the SASP, a pro-inflammatory secretome comprising interleukin-6 (IL-6), interleukin-8 (IL-8), and matrix metalloproteinases—can promote tumor progression by creating an immunosuppressive microenvironment by recruiting regulatory T cells and inhibiting T cells functions, enhancing invasion, and facilitating angiogenesis [31, 32]. This duality underscores the therapeutic complexity of senescence: senolytic therapies aim to eliminate SCs to mitigate SASP-driven tumor progression, while inducing senescence in cancer cells remains a strategy to curb tumor growth. The role of senescence in cancer thus reflects a delicate balance between protective and deleterious effects, modulated by context, disease stage, and microenvironmental factors.

A significant barrier to effective SCs clearance is their resistance to apoptosis, driven by altered expression of pro-apoptotic and anti-apoptotic factors [33], which limits the efficacy of senolytic therapies. Despite their potential, senolytics targeting apoptosis are hindered by the heterogeneity of SCs populations, off-target effects, and incomplete clearance [34]. Furthermore, the accumulation of iron in SCs may exacerbate fibrosis and perpetuate senescence upon release during apoptosis [35, 36]. Consequently, there is a pressing need for alternative therapeutic strategies that selectively target SCs while minimizing collateral tissue damage.

## 3. Overview of Ferroptosis:

Ferroptosis, first described by Dixon [14], is a caspase-independent form of programmed cell death driven by iron-dependent lipid peroxidation, distinct from apoptosis and necrosis. In SCs, heightened susceptibility to ferroptosis arises from elevated divalent iron (Fe^2+^) due to dysregulated ferritinophagy and increased PUFA content in cellular membranes [37]. While oxidative stress is a prerequisite for ferroptosis, its induction requires the specific failure of antioxidant defenses, such as glutathione peroxidase 4 (GPX4), which neutralizes lipid hydroperoxides using glutathione (GSH) [15]. Although general ferroptosis mechanisms, including iron metabolism and GSH synthesis, are comprehensively reviewed elsewhere [38], this discussion focuses on the unique vulnerabilities of SCs.

Iron accumulation in senescent cells, driven by altered dynamics of ferritin and transferrin receptor 1 (TFR1), catalyzes reactive oxygen species (ROS) production via the Fenton reaction, amplifying lipid peroxidation [39]. Various hallmarks of ferroptsis are shown in figure 1. This process is further enhanced by increased lipoxygenase (LOX) activity and reduced GPX4 levels in senescent cells [40]. Unlike cancer cells, which exhibit variable ferroptosis resistance, the consistently iron-rich state of SCs provides a stable therapeutic window for ferroptosis-based interventions [18, 41].

Ferroptosis is characterized by the accumulation of lipid peroxides, leading to membrane damage and cell death. Iron overload triggers the Fenton reaction, generating ROS that induce lipid peroxidation and compromise cell membrane integrity [15, 42]. Distinct from apoptosis (Fig. 2), necrosis, and autophagy, ferroptosis is caspase-independent and does not rely on BCL-2 family proteins. It is primarily driven by the peroxidation of PUFAs, particularly within cell membranes [43]. Antioxidant systems, such as the ferroptosis suppressor protein 1 (FSP1)-coenzyme Q10 (CoQ10)-NAD(P)H axis, mitigate lipid peroxidation under normal conditions, preventing ferroptosis [44, 45].

**Figure 1.**
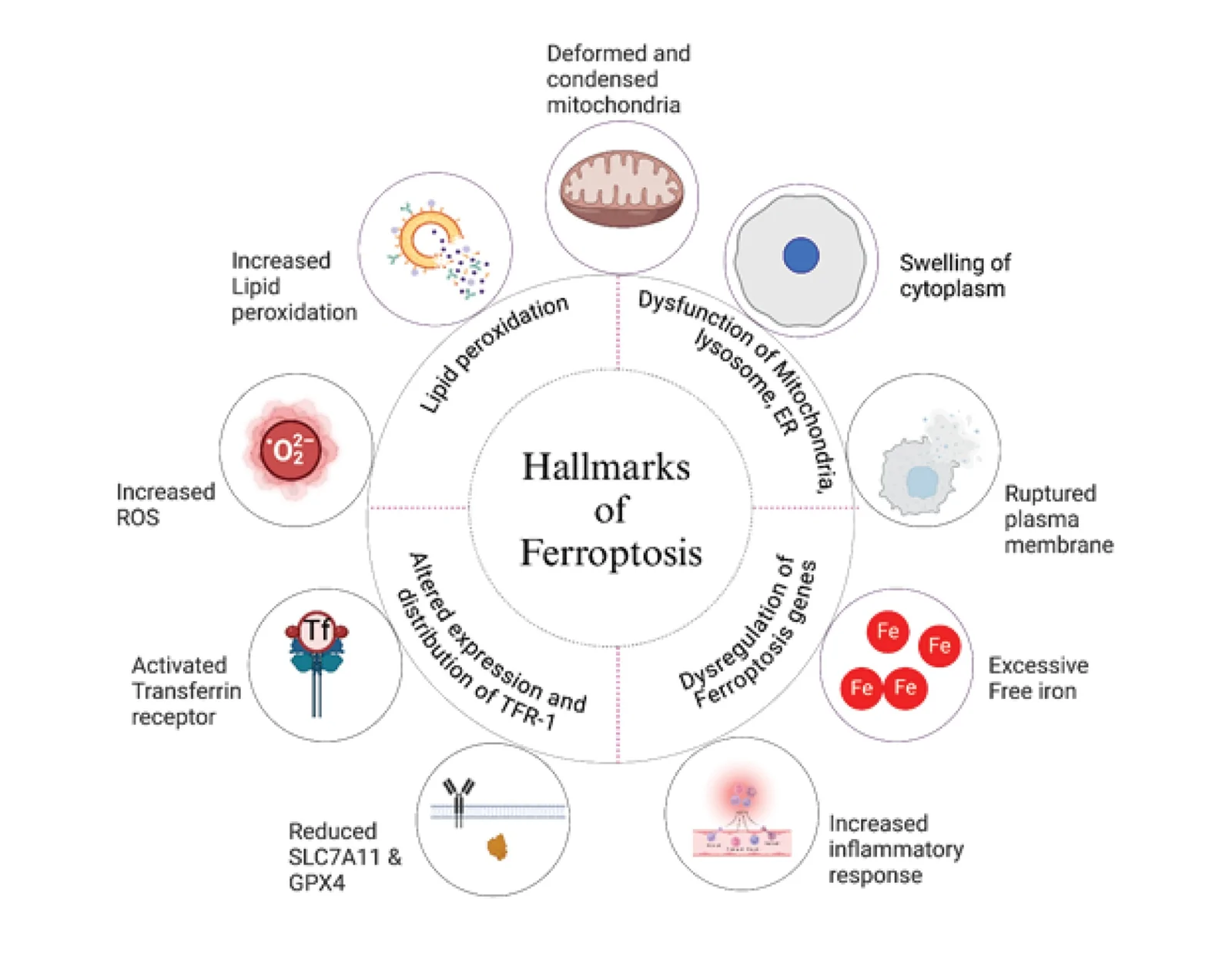
**Hallmarks of Ferroptosis**. Increase in ROS, deformed mitochondria, ruptured plasma membrane and increased inflammatory response overlapped with the hallmarks of senescence.

Oxidative stress, marked by elevated ROS levels, is a hallmark of SCs and a prerequisite for ferroptosis. However, ferroptosis does not occur indiscriminately and requires specific conditions, including iron-dependent lipid peroxidation and GPX4 inhibition [14]. In SCs, oxidative stress may alternatively lead to apoptosis via caspase activation or necrosis via mitochondrial rupture, depending on factors such as iron availability and antioxidant capacity. Thus, distinguishing ferroptosis from other oxidative stress-induced outcomes in senescent cells necessitates the use of specific markers, such as lipid ROS levels or rescue with ferrostatin-1.

Both cancer and SCs, characterized by iron accumulation, exhibit heightened susceptibility to ferroptosis. Similarly, aged tissues frequently display iron overload, underscoring the therapeutic potential of ferroptosis modulation in addressing cancer and age-related diseases [46]. Nevertheless, translating ferroptosis-based therapies into clinical practice presents challenges, particularly the need for selective inducers that target pathological cells without disrupting normal tissue homeostasis.

### 3.1 Iron Metabolism

Iron is integral to cellular physiology due to its electron-donating properties, which are critical for enzymatic reactions and redox homeostasis. In ferroptosis, an iron-dependent form of programmed cell death, the accumulation of ferrous iron (Fe^2+^) plays a pivotal role in driving lipid peroxidation and subsequent cell demise ([47, 48]. Fe^2+^ undergoes oxidation through the Fenton reaction, generating hydroxyl radicals and peroxides that initiate lipid peroxidation, compromising membrane integrity and culminating in cell death [47].

**Figure 1.**
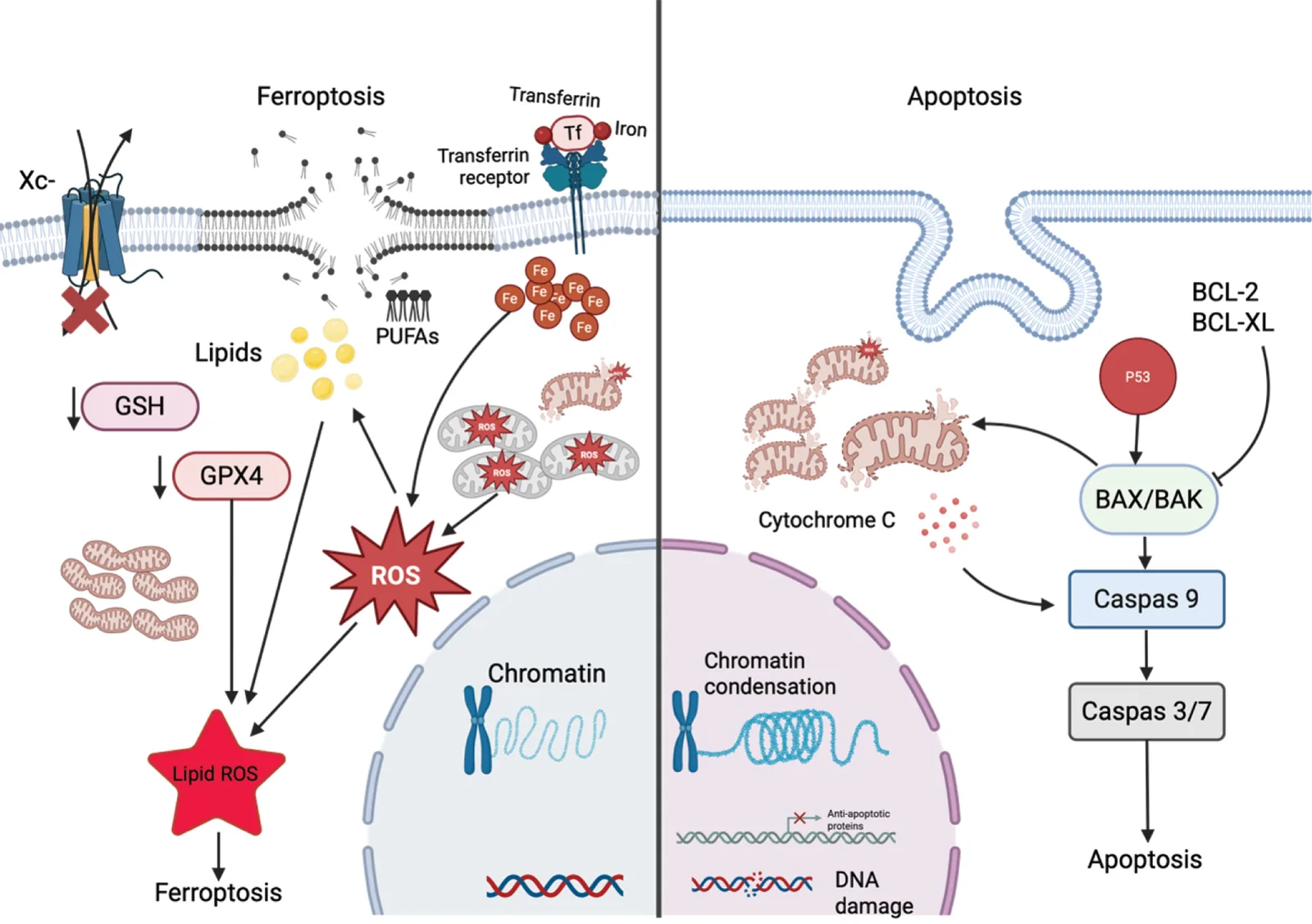
**Non-apoptotic and apoptotic cell death**. This schematic compares ferroptosis (left) and apoptosis (right) in SC. Ferroptosis involves Xc^-^ inhibition, reducing GSH and GPX4, leading to lipid ROS accumulation via iron (Fe) and mitochondrial ROS, causing membrane damage without chromatin condensation. Apoptosis, triggered by DNA damage, activates BAX/BAK, releasing cytochrome c and engages caspases 9 and 3/7, resulting in chromatin condensation. SCs resist apoptosis (BCL-2/BCL-XL upregulation) but are ferroptosis-prone (high ROS).

The uptake of Fe^2+^ is facilitated by divalent metal transporter 1 (DMT1), while its storage within ferritin is tightly regulated by transferrin receptor 1 (TFR1) to maintain cellular iron homeostasis [47]. Dysregulation of these processes, particularly the dysfunction of ferritin and solute carrier family 7 member 11 (SLC7A11) a component of the cystine/glutamate antiporter system Xc^-^ which leads to Fe^2+^ accumulation, significantly enhancing ferroptosis susceptibility [39]. SCs have altered iron metabolism, and dysfunctional autophagy leads to inhibit ferritin and to release iron, thus promoting accumulation of iron due to reduced clearance of stored iron. Transferrin 1 receptor persistently uptake iron which further enforces iron overload in SCs. and allows TfR1 to store iron [49, 50]. This iron overload catalyzes reactive oxygen species (ROS) production, amplifying lipid peroxidation through interactions with PUFAs in cellular membranes [43]. Furthermore, the downregulation of antioxidant defenses, such as glutathione peroxidase 4 (GPX4), exacerbates ferroptosis vulnerability by failing to neutralize lipid hydroperoxides [51]. Recent studies also highlight the role of ferritinophagy-related proteins, such as nuclear receptor coactivator 4 (NCOA4), in mediating iron release from ferritin, further contributing to ferroptosis in SCs [35]. These findings underscore the therapeutic potential of targeting iron metabolism to selectively induce ferroptosis in pathological cells while preserving normal tissue function, though challenges remain in developing precise modulators to avoid off-target effects.

### 3.2 Lipid Metabolism

Lipid metabolism plays a central role in ferroptosis, a programmed cell death pathway driven by iron-dependent lipid peroxidation. Polyunsaturated fatty acids (PUFAs), abundant in cellular membranes, are highly susceptible to oxidation by reactive oxygen species (ROS), generating toxic lipid peroxides, such as 4-hydroxynonenal (4-HNE) and malondialdehyde (MDA), which precipitate ferroptotic cell death [43, 52]. Enzymes such as acyl-CoA synthetase long-chain family member 4 (ACSL4) and lysophosphatidylcholine acyltransferase 3 (LPCAT3) catalyze the incorporation and modification of PUFAs into phospholipids, amplifying lipid peroxidation and promoting ferroptosis [51]. Inhibition of these enzymes has been shown to suppress ferroptosis, highlighting their critical regulatory role. In SCs, elevated PUFA content, coupled with dysregulated lipid homeostasis, enhances ferroptotic susceptibility, particularly under conditions of iron overload and oxidative stress [37]. Additionally, lipoxygenases (LOXs), notably arachidonate 15-lipoxygenase (ALOX15), contribute to PUFA oxidation, further driving lipid peroxide accumulation [40]. The interplay between lipid metabolism and antioxidant defenses, such as glutathione peroxidase 4 (GPX4), is pivotal, as GPX4 mitigates lipid peroxidation by reducing hydroperoxides, and its inhibition accelerates ferroptosis [15]. Recent studies also implicate lipid droplet accumulation in SCs as a modulator of ferroptosis sensitivity, potentially serving as a reservoir for oxidizable lipids [35]. These findings underscore the therapeutic potential of targeting lipid metabolism to selectively induce ferroptosis in pathological cells. However, challenges remain in developing specific inhibitors of ACSL4, LPCAT3, or LOXs to avoid disrupting normal lipid homeostasis, necessitating further research to optimize ferroptosis-based interventions

### 3.3 Antioxidant Regulation

Antioxidant systems play a critical role in regulating ferroptosis, a form of programmed cell death driven by iron-dependent lipid peroxidation. Glutathione peroxidase 4 (GPX4), in concert with ferroptosis suppressor protein 1 (FSP1), mitigates lipid peroxidation by converting lipid hydroperoxides into non-toxic alcohols, thereby preventing ferroptotic cell death [44, 45]. Coenzyme Q10 (CoQ10), a key component of the FSP1-CoQ10-NAD(P)H axis, serves as a potent lipid-soluble antioxidant, neutralizing peroxyl radicals in cellular membranes and reinforcing anti-ferroptotic defenses [44]. Additionally, dihydroorotate dehydro-genases (DHODHs) contribute to antioxidant protection by supporting mitochondrial stability and CoQ10 biosynthesis, further inhibiting ferroptosis [53]. In SCs, compromised antioxidant defenses, including reduced GPX4 expression and glutathione (GSH) depletion, heighten susceptibility to ferroptosis, particularly under conditions of iron overload and elevated reactive oxygen species (ROS) [37]. The nuclear factor erythroid 2-related factor 2 (Nrf2) pathway, a master regulator of antioxidant gene expression, modulates ferroptosis sensitivity by upregulating GPX4, SLC7A11, and heme oxygenase-1 (HO-1), which collectively counteract lipid peroxidation [54]. Dysregulation of Nrf2 in SCs, coupled with increased oxidative stress, creates a permissive environment for ferroptosis [41]. Recent studies also highlight the role of system Xc^-^, which imports cystine for GSH synthesis, in maintaining antioxidant balance, with its inhibition by agents like erastin triggering ferroptosis [14]. These findings underscore the therapeutic potential of targeting antioxidant pathways to selectively induce ferroptosis in pathological cells. However, achieving specificity without disrupting normal cellular redox homeostasis remains a key challenge for clinical translation.

### 3.4 Ion Channels and Glutathione Metabolism

Ion channels and glutathione (GSH) metabolism are pivotal regulators of ferroptosis, an iron-dependent form of programmed cell death. Mechanosensitive ion channels, such as Piezo1, modulate iron overload and ferroptosis by facilitating calcium influx, which amplifies reactive oxygen species (ROS) production and lipid peroxidation [55, 56]. GSH, a critical antioxidant, supports ferroptosis suppression by activating glutathione peroxidase 4 (GPX4), which neutralizes lipid hydroperoxides and mitigates ROS-induced damage [57, 58]. The uptake of cysteine, a rate-limiting precursor for GSH synthesis, is mediated by the cystine/glutamate antiporter system Xc^-^, which plays an essential role in maintaining cellular redox balance and inhibiting ferroptosis [57]. In SCs, dysregulation of system Xc^-^ and reduced GSH levels heighten ferroptotic susceptibility, particularly in the context of iron accumulation and oxidative stress [37]. Inhibition of system Xc^-^ by agents like erastin disrupts GSH synthesis, triggering ferroptosis by depleting GPX4 activity [14]. Additionally, voltage-dependent anion channels (VDACs) regulate mitochondrial iron uptake and ROS generation, further influencing ferroptosis sensitivity [59]. Recent studies also highlight the role of transient receptor potential (TRP) channels in modulating calcium dynamics and oxidative stress, linking ion channel activity to ferroptosis in SCs [60, 61]. These findings suggest that targeting ion channels and GSH metabolism offers therapeutic potential for inducing ferroptosis in pathological cells. However, achieving specificity to avoid disrupting normal cellular functions remains a critical challenge for clinical applications.

### 3.5 Induction, Inhibition, and assays

The field of ferroptosis research has witnessed remarkable progress, marked by the development of novel activators and inhibitors that target its intricate regulatory mechanisms. As an iron-dependent form of programmed cell death, ferroptosis is governed by a delicate interplay of lipid peroxidation, iron metabolism, and antioxidant defenses, offering unique opportunities for therapeutic intervention, particularly in the context of SCs. Suppression of ferroptosis can be achieved through multiple strategies, including the inhibition of cytoplasmic and mitochondrial reactive oxygen species (ROS), modulation of membrane ion channels, attenuation of lipid peroxidation, and enhancement of glutathione peroxidase 4 (GPX4) and glutathione (GSH) levels. Comprehensive reviews of ferroptosis inhibitors are available elsewhere [62-64]. Additional approaches include inhibiting ferritinophagy to limit iron release, downregulating ferroptosis-associated genes, and employing iron chelators to reduce free iron availability.

Pharmacological inducers, such as erastin, RSL3, FIN56, and sulfasalazine, have demonstrated efficacy in sensitizing cells to ferroptosis in vitro by disrupting antioxidant defenses or exacerbating lipid peroxidation. Erastin, for instance, inhibits system Xc^-^, impairing cysteine uptake and GSH synthesis, while RSL3 directly targets GPX4, amplifying lipid hydroperoxide accumulation. Conversely, inhibitors like ferrostatin-1 (Fer-1), liproxstatin-1 (Lip-1), deferoxamine (DFO), and α-tocopherol (vitamin E) are widely utilized to suppress ferroptosis cell death without affecting proliferating cells. These agents exert their protective effects through potent antioxidant properties, scavenging lipid radicals, and chelating free iron, thereby mitigating the oxidative cascade central to ferroptosis.

The antioxidant capacity of these inhibitors, particularly in neutralizing lipid peroxyl radicals and sequestering reactive iron, is fundamental to their anti-ferroptosis activity. Upregulation of antioxidant genes, notably GPX4 and ferroptosis suppressor protein 1 (FSP1), constitutes another critical mechanism for ferroptosis inhibition. GPX4, in concert with GSH, reduces lipid hydroperoxides to non-toxic alcohols, while FSP1 supports CoQ10-mediated antioxidant defenses. Disruption of the GPX4, FSP1, system Xc^-^, or GSH pathways markedly promotes ferroptosis in cellular models, underscoring their regulatory significance. For a detailed review of ferroptosis inducers, readers are directed to Ma et al.[65]. Recent evidence suggests that targeting multiple pathways concurrently such as iron metabolism and lipid peroxidation enhances ferroptosis induction in SCs, capitalizing on their iron-rich microenvironment and heightened oxidative stress [66].

Assessment of ferroptosis encompasses a suite of molecular and biochemical markers to capture its multifaceted nature. In vitro studies typically measure cell viability, intra- and extracellular free iron levels, cytoplasmic and mitochondrial ROS, GPX4 and GSH concentrations, glutamate release, cysteine uptake, and nicotinamide adenine dinucleotide phosphate (NADPH) levels. These parameters provide a comprehensive view of ferroptotic dynamics, reflecting perturbations in iron homeostasis, redox balance, and lipid metabolism. In vivo evaluations often focus on the expression of prostaglandin-endoperoxide synthase 1 (PTGS1) and 2 (PTGS2), enzymes intricately linked to lipid peroxidation and iron metabolism, which serve as reliable indicators of ferroptosis activation [66].

Reflecting on four decades of research, it is evident that ferroptosis represents a promising frontier for selectively targeting SCs in aging and age-related diseases. However, the complexity of its regulation demands precision in therapeutic design to avoid off-target effects while maximizing efficacy. Future investigations should prioritize the development of synergistic approaches that integrate pathway-specific modulators, ensuring robust ferroptosis induction in pathological contexts without compromising physiological homeostasis.

## 4. Senescence Regulation by Ferroptosis

Ferroptosis, an iron-dependent form of programmed cell death, and cellular senescence, marked by cell cycle arrest, converge through shared molecular pathways that drive aging and age-related diseases (Table 1). SCs accumulate in tissues, fueling pathologies like cancer, neurodegeneration, and fibrosis. Their iron-rich microenvironment and disrupted lipid homeostasis make them uniquely susceptible to ferroptosis, offering a transformative senolytic strategy that bypasses the limitations of apoptosis-based therapies [49, 67]. Understanding this bidirectional interplay where ferroptosis can trigger senescence and SCs are primed for ferroptosis is critical for developing precision therapeutics.

A pivotal link between ferroptosis and senescence is proteostasis, the delicate balance of protein synthesis, folding, and degradation that collapses in SCs. Impaired proteostasis leads to misfolded protein accumulation and oxidative stress, creating a fertile ground for ferroptosis. The transcription factor nuclear respiratory factor 1 (NRF1) regulates this nexus by modulating proteasome activity. Kotschi and coworkers showed that NRF1 activation clears damaged proteins [68], reducing ferroptosis, while Li et al. (2024) found that NRF1 suppression in stressed cells amplifies lipid peroxidation, enhancing ferroptosis susceptibility [69]. The transcription factor nuclear factor erythroid-2, like-1 (NFE2L1) has been shown to involve in proteosomal activity of ferroptsis process [68] and its interaction with DNA-damage inducible 1 homolog 2 (DDI2) [70] can be used to sensitize SCs to ferroptosis. In SCs, compromised proteostasis tips the balance toward ferroptosis, particularly in iron-laden settings, presenting a therapeutic window.

**Table 1:** Differences between Ferroptosis and Senescence.

Feature	Ferroptosis	Senescence	References
**ROS**	Excessive lipid peroxidation and free iron generate ROS via Fenton’s reaction.	DNA damage, mitochondrial dysfunction, and SASP contribute to ROS production.	[14, 19]
**P53**	P53 regulates SLC7A11, enabling cysteine uptake, which decreases glutathione levels and triggers ferroptosis.	DNA damage activates p53, leading to p21 activation and promoting SASP.	[150-152]
**Inflammation**	Ferroptotic cells release DAMPs and inflammatory mediators, exacerbating tissue damage.	Low-grade inflammatory secretion from SCs contributes to aging and the development of SASP.	[153, 154]
**Oxidative Stress**	Excessive ROS and free iron drive lipid peroxidation, resulting in oxidative stress.	ROS and mitochondrial dysfunction are key drivers of oxidative stress in senescence.	[155, 156]
**Sterols**	Sterols and PUFAs contribute to lipid peroxidation, influencing ferroptosis susceptibility.	Sterols are vital components of cell membranes and lipid metabolism. Altered lipid profiles contribute to inflammation and metabolic dysfunction.	[37, 157]
**Iron Accumulation**	Altered iron metabolism is a key trigger for ferroptosis, promoting oxidative damage.	SCs accumulate 30-fold more free iron than young cells, increasing susceptibility to oxidative stress.	[15, 49]
**Macrophages**	Macrophages clear ferroptotic cells and secrete DAMPs, which modulate tissue inflammation.	Macrophages clear SCs, though excessive activation leads to chronic inflammation.	[158, 159]
**Mitochondria**	Iron accumulation in mitochondria generates ROS and disrupts membrane integrity.	Mitochondrial dysfunction in SCs releases ROS and cytochrome C, promoting inflammation and cell death.	[155, 156, 160]
**Lysosomes**	Lysosomes mediate ferritinophagy, releasing free iron to trigger ferroptosis.	Defective autophagy in SCs leads to the accumulation of autophagic granules, contributing to metabolic dysfunction.	[36, 49, 119, 161]
**ER Stress**	ER stress activates the C/EBP pathway, increasing ROS and lipid peroxidation.	Persistent ER stress in SCs activates the unfolded protein response (UPR), promoting ROS production and SASP.	[162, 163]
**Nrf2**	Oxidative stress reduces Nrf2-mediated activation of antioxidant pathways, exacerbating ferroptosis.	Nrf2 activation declines with age, impairing antioxidant defenses and contributing to SASP.	[54, 95, 98]
**c-GAS-STING**	ROS activate c-GAS-STING signaling, downregulating Nrf2 and exacerbating oxidative stress.	Damaged DNA detected by c-GAS activates STING and induces SASP via interferon-1 signaling.	[164, 165]
**NF-kB Pathway**	NF-kB activation in ferroptotic cells amplifies inflammation and tissue dysfunction.	In SCs, NF-kB activation induces SASP, contributing to chronic inflammation.	[166]
**Differences between the ferroptosis and Senescence**			
**Cell State**	Irreversible, regulated form of cell death.	Cell proliferation ceases; cells remain viable and metabolically active, with potential reversibility.	[37, 167]
**Mechanism**	Triggered by iron-dependent lipid peroxidation and dysregulated GSH/GPX4 levels.	Caused by various stresses and leads to the development of the senescence-associated secretory phenotype (SASP).	[15, 26]
**Morphology**	Characterized by swollen, ruptured membranes.	Cells appear flattened, enlarged, and exhibit altered morphology.	[168, 169]
**Biological Functions**	Clears unwanted or damaged cells.	Contributes to wound healing, tumor suppression, and tissue dysfunction.	[170, 171]
**Therapeutic Interventions**	Ferroptosis inducers or inhibitors.	Senolytic and senomorphic agents.	[172, 173]

This molecular convergence opens innovative avenues. Modulating proteostasis with NRF1 agonists or proteasome enhancers could fine-tune ferroptosis sensitivity, enabling targeted SC clearance. For instance, NRF1 modulation in neurodegenerative models reduces SC driven inflammation [71]. Combining ferroptosis inducers with proteostasis modulators or senolytics, such as navitoclax, could amplify efficacy across diseases like fibrosis or cancer. Ferroptosis-mediated SC clearance from aged donor organs also holds promise for improving transplant viability, addressing global organ shortages.

However, challenges remain, including SC heterogeneity, potential off-target effects, and the need for robust preclinical models. Advanced proteomics, single-cell analyses, and selective ferroptosis inducers tailored to SC-specific vulnerabilities (e.g., ferritinophagy) will be crucial. By harnessing the senescence-ferroptosis nexus, these strategies could redefine aging therapeutics, but rigorous validation is essential to ensure safety and efficacy

### 4.1 Limitations of Traditional Senolytics

Traditional senolytics, designed to clear SCs via apoptosis, face significant hurdles that curb their therapeutic promise. Drugs like navitoclax and the dasatinib-quercetin combination target SCs but grapple with off-target toxicity, resistance, and incomplete efficacy [72]. For instance, navitoclax, a BCL-2 inhibitor, is linked to thrombocytopenia, limiting its clinical use [73]. Resistance, driven by upregulated BCL-2 family proteins, further undermines effectiveness, while the heterogeneity of SC populations varying by tissue, induction mechanism, and disease context leads to inconsistent clearance [74]. These challenges highlight the urgent need for alternative strategies that exploit SC-specific vulnerabilities beyond apoptotic pathways.

Efforts to improve senolytic specificity have yielded innovative approaches. SCs, marked by elevated β-galactosidase activity, can be targeted using silica nanoparticles or CD9-coated antibodies, which enhance precision [75, 76]. Photodynamic therapy (PDT), which generates reactive oxygen species (ROS) via photosensitive drugs, also shows promise for SC elimination [77, 78]. Yet, these advances do not fully resolve the core issues. Off-target effects persist, as seen with navitoclax’s hematological toxicity, and resistance mechanisms, such as BCL-XL upregulation, continue to thwart complete SC clearance [79]. Moreover, the diverse molecular profiles of SCs spanning fibroblasts in fibrotic lungs to microglia in neurodegenerative brains [80] complicate uniform targeting.

The limitations of apoptosis-based senolytics underscore a critical gap in aging therapeutics. Their reliance on pathways prone to resistance and toxicity calls for novel approaches, such as ferroptosis-based senolytics, which leverage SCs’ iron-rich, oxidative microenvironment. Developing therapies that bypass apoptotic resistance while ensuring specificity will be essential to unlock effective treatments for age-related diseases

### 4.2 SCs are susceptible to Ferroptosis

SCs are vulnerable to ferroptosis, which offers a transformative senolytics strategy. Unlike apoptosis-based senolytics, which falter against SCs’ anti-apoptotic defenses, ferroptosis exploits their iron-rich microenvironment, elevated PUFAs, and dysregulated lipid metabolism to induce precise cell death [37]. This susceptibility positions ferroptosis as a compelling alternative for clearing SCs in conditions like cancer, neurodegeneration, and fibrosis.

SCs accumulate iron through disrupted homeostasis, driven by altered ferritinophagy and transferrin receptor activity, creating a fertile ground for ferroptosis [49]. Elevated PUFA levels, coupled with increased lipoxygenase activity, amplify lipid peroxidation a critical ferroptosis driver making SCs exquisitely sensitive to this process [40]. Ferroptosis inducers like erastin capitalize on these vulnerabilities, demonstrating efficacy in preclinical models of resistant SCs, where apoptosis fails [81]. For instance, erastin triggers lipid peroxidation in senescent fibroblasts, bypassing BCL-2 upregulation to achieve robust clearance [82].

The therapeutic potential of ferroptosis extends beyond standalone induction. Combining ferroptosis inducers with immune modulators enhances specificity and efficacy, leveraging immune surveillance to clear SCs and mitigate the SASP in age-related diseases [66]. In cancer models, this synergy disrupts SC-driven tumor microenvironments, while in neurodegenerative contexts, it reduces inflammation.

Realizing this potential requires overcoming challenges, such as SC heterogeneity and off-target effects of inducers like erastin. Advanced approaches, including nanoparticle-based delivery and single-cell analyses, are refining selectivity by targeting SC-specific lipid profiles. By harnessing the molecular vulnerabilities of SCs iron accumulation, PUFA enrichment, and lipid peroxidation ferroptosis offers a paradigm shift in senolytics therapy. Rigorous preclinical validation and biomarker development (4-HNE levels) will be essential to translate these insights into safe, effective treatments for aging-related pathologies.

### 4.3 Elimination of SCs by Ferroptosis

SCs, drivers of aging and diseases like cancer, neurodegeneration, and fibrosis, are prime targets for ferroptosis, an iron-dependent form of programmed cell death. Their dysregulated iron metabolism, elevated polyunsaturated fatty acids, and heightened lipid peroxidation render SCs uniquely susceptible to ferroptosis, offering a potent senolytics strategy that bypasses apoptosis resistance. Over fifteen studies highlight ferroptosis’s efficacy in clearing SCs, with profound implications for age-related pathologies (Table 2).

**Table 2:** Summary of studies: Elimination of SCs by ferroptosis.

Experimental Model	Interventions	Mechanism of Action	Methods	Implications	References
**Primary endothelial cells, MSCs, IMR90 cells**	TRX-CBI, FIN56	Excess iron accumulation, reduced GPX4	Iron quantification (FerroFarread), lipid peroxidation (C11 Bodipy)	Development of senolytics to treat biological aging	[81]
**Kidney tissue slices, primary tubular epithelial cells**	RSL3, Erastin, FIN56, FINO2	Increased Lipoxygenase-5, reduced GPX4	Lipoxygenase assay, GPX4 (western blot)	Renal disease, removal of SCs from transplanted aged kidneys	[85]
**Human lens epithelial cells, mouse lens epithelium**	Erastin, RSL3	GSH depletion, downregulation of SLC7A11, ferroportin	Free iron assay, GSH assay	Cataract formation, aging lens	[84]
**Human dermal fibroblasts (HDFs)**	JQ1	Downregulation of GPX4, SLC7A11, Nrf2, upregulation of p53	qRT-PCR, BODIPY staining	Development of new senolytics	[83]
**HskM cell model**	Chloroquine (CQ), L-Leucyl-L-Leucine methyl ester (LLOMe)	Disruption of ferritinophagy, NCOA4 knockdown	Malondialdehyde (MDA) assay, lipid peroxidation, free iron	Development of alternative senolytics	[35]
**Huh7 and HEK293T cells**	Magnetic EV-based delivery of iBax mRNA, BAX activator BTSA1	Inducing BAX oligomerization on mitochondrial membrane, apoptosis	NA	Atherosclerosis, safe and efficient senolytics	[174]
**Primary mouse fibroblasts, mouse model**	Fe3O4 encapsulated in PLGA nanospheres, modified with galactose	Fenton-reaction-dependent ferroptosis	Fe+2/+3 ions, intracellular ROS, LPO, GSH measurement	Treatment of diabetic wound healing	[175]
**Human primary chondrocytes, rat OA model**	EAAT1 inhibitor UCPH-101, FINO2	Inhibition of Glu and Glutathione-EAAT1 anti-ferroptosis axis	MDA, GSH, Glu, cysteine, glycine, total iron assay	Delay the progression of osteoarthritis	[91]
**Pancreatic and breast cancer cells**	Palbociclib, I-BET726, JQ1, RSL3	GPX4 inhibition with CDK4/6 and BRD4 inhibitors	LPO, ROS, GSH	Cancer treatment	[89]
**LO2 and 293T cells**	4,4′-dimethoxychalcone (DMC), quercetin, dasatinib	Blocking FTH, activating ferritinophagy, increased free iron	Intracellular free iron, MDA assay	Development of new senolytics for age-related diseases	[88]
**Murine fibroblasts, mouse model**	DHA, Ferrostatin-1, BafA1, DFO	Activating ferritinophagy, downregulation of AMPK & mTOR	ROS, GPX4, PTGS2, FTH, TfR1	Development of new senolytics	[108]
**Mouse skin fibroblasts, mice STZ model**	AMPK activator A769662	AMPK activation, NCOA4-mediated ferritinophagy	MDA, total Fe+2, GPX4, ROS	Treatment for diabetic non-healing wounds	[100]
**Mice liver cells**	GPX4 blocker	Inhibition of GPX4 in SCs	NA	NAFLD/NASH liver diseases	[112]
**B16F10 cells, in vivo**	Ratiometric coencapsulation of Palbociclib, Auranofin in liposomes	Depletion of GSH and NADPH, sensitizing cells to ferroptosis	Lipid peroxidation, MDA assay	Melanoma cancer treatment	[90]
**Skin fibroblasts, cancer cells**	17DMAG-loaded iron oxide nanoparticles functionalized against CD26	Targeting ferritinophagy and apoptosis	NOCA4 and ferritin expression	Lung and skin SCs, cancer SCs	[92]
**Brain aging, neurodegenerative diseases**	CK, Fer-1, Necrostatin-1, Z-VAD-FMK	Ginsenoside compound K (CK) inhibits ferroptosis, improves mitochondrial function	NA	Brain aging	[92]

Iron sequestration in lysosome disrupts ferritinophagy via proteins like ferritin heavy chain 1 (FTH1) and nuclear receptor coactivator 4 (NCOA4), and sensitizes SCs to ferroptosis [35]. Pharmacological agents, such as the ferric iron-activated prodrug TRX-CBI and bromodomain inhibitor JQ1, enhance lipid peroxidation, effectively eliminating SCs, though JQ1’s effects await broader validation [81, 83]. In preclinical models, ferroptosis inducers like erastin and RSL3 target SCs with reduced glutathione peroxidase 4 (GPX4) or increased lipoxygenase activity, as seen in cataract lens epithelial cells and kidney epithelia [84, 85].

Advanced delivery systems, such as Fe3O4 nanoparticles and iBax mRNA-loaded nanoparticles, improve precision, promote ferroptosis in SCs for conditions like diabetic wounds and osteoarthritis [86, 87]. Natural compounds, like 4,4′-dimethoxychalcone, also induce ferritinophagy-mediated ferroptosis, expanding therapeutic options [88, 89]. Combination therapies further enhance efficacy: co-treatment with bromodomain-containing protein 4 (BRD4) inhibitors overcomes GPX4-driven resistance in palbociclib-induced SCs, amplifying reactive oxygen species and ferroptosis death [89].

Despite these advances, ferroptosis resistance, driven by antioxidant defenses or excitatory amino acid transporter 1 (EAAT1) overexpression, poses challenges, as seen in non-alcoholic fatty liver disease and melanoma [90, 91]. Optimizing selective inducers, refining nanoparticle-based delivery, and validating combination therapies in robust preclinical models are critical next steps. By harnessing SCs’ molecular vulnerabilities, ferroptosis promises to redefine senolytics therapy, but rigorous clinical translation is essential to ensure safety and efficacy across diverse age-related diseases (Fig. 4).

### 4.4 Ferroptosis Modulation of SCs

Ferroptosis, an iron-dependent, lipid peroxidation-driven form of programmed cell death, exerts a dual influence on cellular senescence, offering innovative strategies to manage aging and age-related diseases. By inducing ferroptosis, SCs drive pathologies like cancer, neurodegeneration, and fibrosis can be selectively eliminated, exploiting their iron-rich microenvironment and lipid dysregulation [81]. Conversely, inhibiting ferroptosis in pre-senescent cells can delay or mitigate senescence by preserving redox homeostasis, reducing oxidative stress, and preventing SC accumulation, presenting a nuanced therapeutic paradigm.

Inhibiting ferroptosis can delay onset of senescence. For instance, ferroptosis inhibitors like ferrostatin-1 attenuate oxidative damage in brain aging models, delaying SC formation by upregulating glutathione peroxidase 4 (GPX4) via nuclear factor erythroid 2-related factor 2 (Nrf2) signaling [92]. Similarly, Klotho protein protects renal and myocardial cells from senescence by modulating oxidative stress pathway [93]. In tendon stem cells, platelet-derived exosomes mitigate senescence through the AMP-activated protein kinase (AMPK)/Nrf2/GPX4 axis, enhancing regenerative potential [94, 95]. Pharmacological agents, such as sennoside A and rutin, reduce ferroptosis-induced oxidative damage in Alzheimer’s and ovarian aging models, respectively, via Nrf2/heme oxygenase-1 (HO-1) activation [96].

Epigenetic and metabolic regulation further shapes ferroptosis’s anti-senescence potential. Suppression of the elastin (ELN) gene accelerates senescence via histone demethylase activity, but Nrf2 activation reverses this, highlighting iron-dependent epigenetic modifiers as therapeutic targets [97]. In lung adenocarcinoma, NUAK2 depletion amplifies ferroptosis to constrain tumor growth while delaying. Natural compounds like ginsenoside Compound K and selenium enhance mitochondrial function and Nrf2/HO-1 signaling, mitigating neuronal and intervertebral disc senescence [92, 98].

This dual role of eliminating SCs via ferroptosis induction or delaying senescence through inhibition demands mechanistic clarity and robust preclinical models. Optimizing selective inhibitors, leveraging nanoparticle delivery, and exploring epigenetic modulators will be critical to translate these insights into therapies for aging-related pathologies, balancing efficacy with safety (Table 3).

**Table 3:** Summary of studies: Alleviation of SCs and aging by ferroptosis.

Experimental Model	Interventions	Mechanism of Action	Methods	Implications	References
**Human CRC and cell lines**	Overexpression of RSLD1, MG-132, DFO	RSLD1 regulates FTH1 and iron accumulation via mRNA stability	Free iron, GSH, GPX4 assays	Cancer treatment	[99]
**293T and cell lines**	FDX2-KO, GPX4 inhibitor, ML-162, iFSP1	FDX2-KO induces ferroptosis via p53	NA	Ovarian cancer cells	[176]
**C. elegans**	Nanoplastics and benzo[a]pyrene	Ferroptosis causes senescence, accelerating aging via mitochondrial integrity disruption	NA	Aging	[93]
**Mouse model of CIRI**	Lentiviral GRSF1, Ferrostatin-1, RSL3	GRSF1 blocks ferroptosis and iron metabolism, elevates GPX4	Fe+2, Fer, IRP2, TfR1, MDA, SOD, GSH	Cerebral ischemia-reperfusion injury (CIRI)	[96]
**Granulosa cells and SWFs**	Rutin, Erastin, ML385, DMF (Nrf2 activator)	Rutin upregulates Nrf2/HO-1, blocking ferroptosis and oxidative stress	HO-1, Nrf2, Fe+2, GPX4, MDA, SOD, GSH, T-AOC	Ovarian aging	[177]
**CSSCs**	Levofloxacin, Ferrostatin-1, Lov	Ferroptosis inhibitors reduce inflammation and fibrosis	GPX4, SLC7A11, ROS, Fe+2	Corneal scarring, bacterial keratitis (BK)	[94]
**H9C2 cells and BalbC mice**	D-gal, Klotho	Klotho reduces oxidative stress via p53/GPX4/SLC7A11, blocks ferroptosis	ROS, SOD, MDA, GSH	Cardiac fibroblast aging	[178]
**Tendon-derived stem cells, Sprague-Dawley rats**	Platelet-derived exosomes, RSL3	Inhibition of ferroptosis via upregulation of GPX4/Nrf2/AMPK	GPX4, lipid peroxidation, GSH	Tendon aging and degeneration	[95]
**MEFs, MRC5**	Histone PHF8 demethylase	Iron chelation reverses ELN-downregulation-induced senescence	PHF8 chromatin chip, MitoROS, ICP-MS	Age-related diseases	[97]
**Mouse, lung epithelial, and cancer cells**	Fer-1	Ferroptosis inhibition alleviates senescence via NAUKA	FTH1, ASCL4, TBARS, GPX4, SLC7A11	Tumor progression, lung adenocarcinoma	[179]
**Mice, osteocytes MLO-Y4**	Eldecalcitol (ED-71)	Vitamin D analog reduces senescence markers and restores ferroptosis markers (Nrf2, 4-HNE)	GPX4, Nrf2, MDA, Fe+2, GSH	Osteoporosis	[180]
**HK-2 cells**	Klotho, ABI3BP inhibition	KO of ABI3BP gene suppresses ferroptosis, alleviates renal aging	GPX4, total iron ions, lipid peroxidation, MDA	Kidney aging	[20]
**Mice, VMCs**	Lipoxstain-1, Overexpression of GPX4	PPARγ activation blocks ferritinophagy, overexpression of GPX4 alleviates senescence	OS, NAD+, GSH, ferritinophagy assay	Age-associated cardiovascular disease	[181]
**Mice and HEI-OCI cells**	Overexpression of KCNMA1 or adenovirus	KCNMA1 downregulation accelerates aging, protects from senescence	NA	Age-related hearing loss	[182]
**Liver fibrosis**	Curcumol	Curcumol increases labile iron pool via iron chelation, reversing senescence	NA	Liver fibrosis	[101]
**Mouse AT2 cells, in vivo**	SIRT3 agonist, Melatonin	SIRT3 overexpression mitigates fibrosis, prevents senescence via melatonin activation	NA	Lung fibrosis and injury	[183]
**Granulosa cells, mice**	KL201, Cry1 stabilizer	KD of NCOA4 delays/reverses senescence by inhibiting ferritinophagy	NA	Age-related female fertility decline	[113]
**Immortalized MEFs, mice**	BACH1, ferroptosis inducer	BACH1 induces FGF21 secretion, blocks ferroptotic stress, reduces GSH, increases Fe+2	NA	Anti-aging, obesity, diabetes, reversal of senescence	[184]
**Mice, HTT22 cells**	VD metabolite 1,25(OH)2D3	VDR/Nrf2/HO-1 axis suppression of ferroptosis, reversal of senescence	ACSL4, MDA, GPX4, HO-1, Nrf2, ALOX15, free iron	Age-related neurodegenerative diseases	[98]
**Nucleus pulposus (NP) cells, human tissue**	Selenium supplementation, ML201, GPX4 blocker	Overexpression of SelK attenuates ferroptosis and aging	NA	Intervertebral disc degeneration (IVDD)	[185]
**Mouse tissue**	GGPP	GGPP reverses senescence, SASP, lipid peroxidation	Fe+2, GSH, GPX4, MDA	Cardiovascular diseases	[186]
**C. elegans**	CeFRH-1	Limiting mitochondrial iron delays aging, activates GPX4, inhibits DGLA	NA	Healthy aging	[187]
**Murine chondrocytes, TBHP model**	DFO, Fer-1	Deferoxamine alleviates chondrocyte senescence, OA progression by iron chelation	NA	Osteoarthritis	[188]

### 4.5 Mechanisms of Ferroptosis in SCs Regulation and Elimination

**Figure 3.**
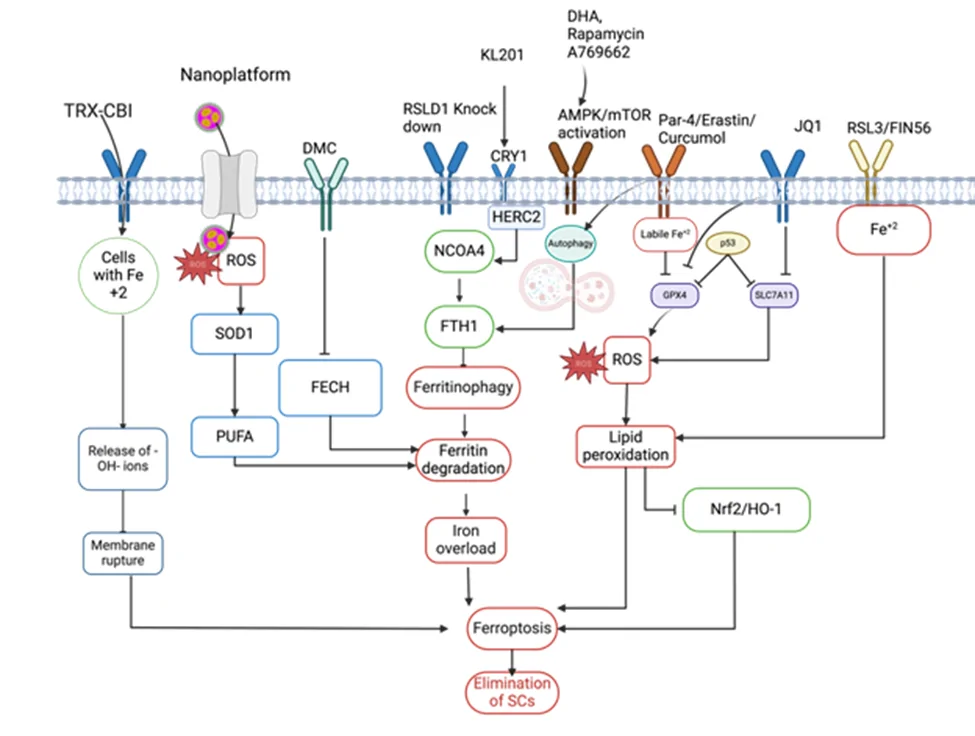
**Pathway for the Elimination of SCs through Ferroptosis**. Figure illustrates the key molecular pathways that have been explored in the literature for eliminating SCs through ferroptosis.

SCs, drivers of aging-related diseases, are primed for ferroptosis—an iron-dependent, lipid peroxidation-driven form of programmed cell death due to their dysregulated iron metabolism and heightened oxidative stress (Fig. 3). Elevated intracellular iron, accumulated through altered ferritinophagy and transferring receptor activity, fuels lipid peroxidation, making SCs vulnerable to targeted elimination. However, SCs use robust counter mechanisms, such as antioxidant defenses and lysosomal iron sequestration, to resist ferroptosis death. Thus, overcoming these limitations is critical for effective senolytics therapies.

Increased PUFAs and lipoxygenase activity drive lipid peroxidation, however, lysosomal sequestration of iron by ferritin complexes limits ferroptosis sensitivity [35]. Disrupting ferritinophagy, a process mediated by ferritin heavy chain 1 (FTH1), ferritin light chain (FTL), and nuclear receptor coactivator 4 (NCOA4) releases lysosomal iron, sensitizing SCs to ferroptosis [99, 100]. Pharmacological agents exploit these vulnerabilities. The ferric iron-activated prodrug TRX-CBI enhances lipid peroxidation, triggering ferroptosis death in SCs, while glutathione peroxidase 4 (GPX4) inhibitors like erastin disrupt antioxidant defenses, amplifying susceptibility [81, 101]. Similarly, the bromodomain inhibitor JQ1 promotes lipid peroxidation in bleomycin-induced senescent fibroblasts, though its broader efficacy requires validation [83].

Advanced delivery systems further refine ferroptosis induction. Engineered nanoparticles, such as those delivering iBax mRNA or Bax activators[102], selectively target SCs, releasing pro-ferroptotic signals to overcome lysosomal barriers. These mechanisms underpin ferroptosis’s dual role in SC regulation: eliminating established SCs by exploiting their iron-rich microenvironment and modulating senescence progression in diseases like cancer and fibrosis [103, 104]. For instance, disrupting FTH1-NCOA4 interactions in senescent fibroblasts enhances ferroptosis clearance, reducing inflammation in preclinical models [105].

Despite these advances, SC heterogeneity and resistance mechanisms, including GPX4 upregulation, pose challenges. Future research must optimize selective inducers and delivery systems, leveraging single-cell analyses to map SC-specific ferroptosis pathways. By targeting iron metabolism and lipid peroxidation, ferroptosis offers a mechanistic cornerstone for senolytics therapies, promising precision in combating age-related pathologies

## 5. Ferroptosis-Mediated Regulation of SCs in Age-Related Diseases:

Ferroptosis, an iron-dependent form of regulated cell death, has emerged as a critical driver of age-related pathologies, including cardiovascular disease, cancer, neurodegenerative disorders, impaired wound healing, female infertility, and bone disorders ([106, 107]. Its intimate link with cellular senescence positions ferroptosis as a promising therapeutic target for eliminating SCs, which accumulate in aging tissues and fuel disease progression. By exploiting the iron-rich microenvironment and lipid dysregulation of SCs, ferroptosis-based strategies offer a novel senolytic approach to mitigate a broad spectrum of age-related conditions.

In diabetic wounds, SC accumulation impairs tissue regeneration, but ferroptosis induction via AMP-activated protein kinase (AMPK) activation and ferritinophagy has shown promise in preclinical models. Nanoparticle-based delivery systems, such as galactose-functionalized Fe3O4 nanoparticles, enhance regenerative outcomes by selectively targeting SCs [100, 108]. Similarly, senescent tendon stem cells, which hinder tissue repair, are amenable to ferroptosis-modulating therapies. Strategies like AMPK activation or platelet-derived exosome therapies reduce oxidative stress and restore regenerative capacity, offering potential for tendon-related disorders [95]. These findings highlight ferroptosis’s role in promoting tissue repair by clearing SCs, with implications for chronic wound management.

**Figure 4.**
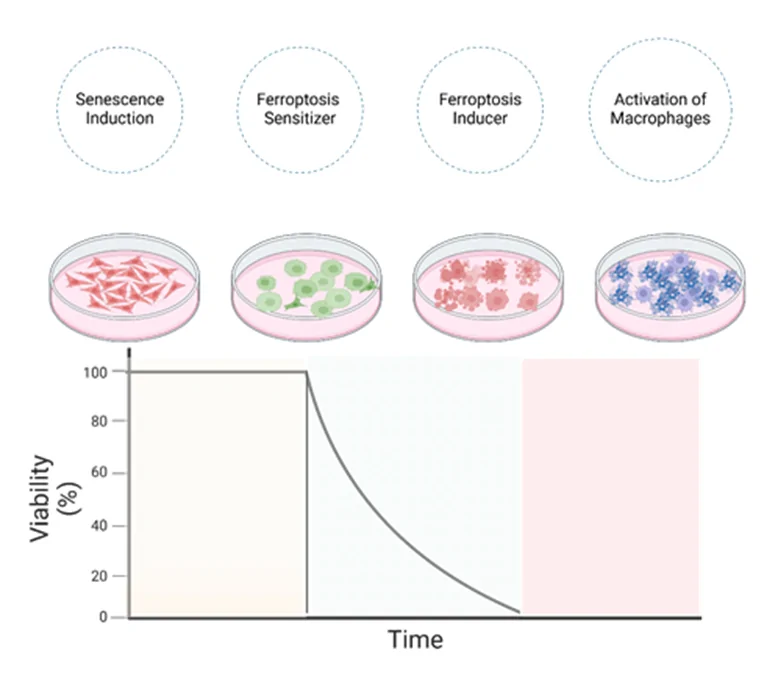
**A modified two-punch strategy for targeted SCs Elimination via Senescence Induction and Ferroptosis-Mediated Clearance**. This schematic illustrates a sequential therapeutic approach to eradicating cancer cells. Initially, senescence is induced in cancer cells (first panel), increasing their susceptibility to ferroptosis through dysregulated iron metabolism and lipid peroxidation. A ferroptosis sensitizer (second panel) primes these SCs by disrupting protective mechanisms, such as lysosomal iron sequestration. Subsequently, a ferroptosis inducer (third panel) triggers iron-dependent programmed cell death, characterized by lipid peroxidation, leading to a decline in cell viability over time (graph below). Finally, activation of macrophages (fourth panel) facilitates the clearance of ferroptotic senescent cancer cells, enhancing therapeutic efficacy. The graph depicts the reduction in cancer cell viability over time, highlighting the synergistic impact of this two-punch strategy in overcoming resistance and promoting selective elimination.

In oncology, ferroptosis presents a dual opportunity. While some cancers, such as melanoma, exhibit ferroptosis resistance [109], these mechanisms can be harnessed in a ‘two-punch’ strategy inducing senescence followed by ferroptosis-mediated SC elimination to enhance treatment efficacy [90]. Combining ferroptosis inducers with senescence-inducing therapies could overcome resistance in malignancies like lung or pancreatic cancer, where SCs drive tumor progression (Fig. 4). This approach underscores ferroptosis’s potential to transform cancer therapeutics by targeting both tumor and SCs populations.

Neurodegenerative disorders also stand to benefit. Ferroptosis contributes to microglial dysfunction in Alzheimer’s and Parkinson’s diseases, where SC accumulation exacerbates neuroinflammation. Targeting ferroptosis in senescent microglia, potentially through iron chelation or lipid peroxidation inhibitors, could mitigate neuronal damage and slow disease progression [92, 110]. These insights pave the way for novel neuroprotective strategies, with preclinical studies in mouse models showing early promise [111].

Beyond these, ferroptosis dysregulation drives pathology in aging organs. In the liver, kidneys, and ovaries, SC accumulation fuels fibrosis, renal dysfunction, and infertility, respectively. Ferroptosis modulation via iron chelation or targeted inducers offers therapeutic potential to restore organ function and delay age-related decline [20, 112, 113]. For instance, iron chelation in ovarian models has improved fertility outcomes by reducing SC-driven oxidative stress (Wu et al., 2023). Additionally, clearing SCs from aged donor organs via ferroptosis could enhance transplant viability, addressing global organ shortages [114].

**Figure 5.**
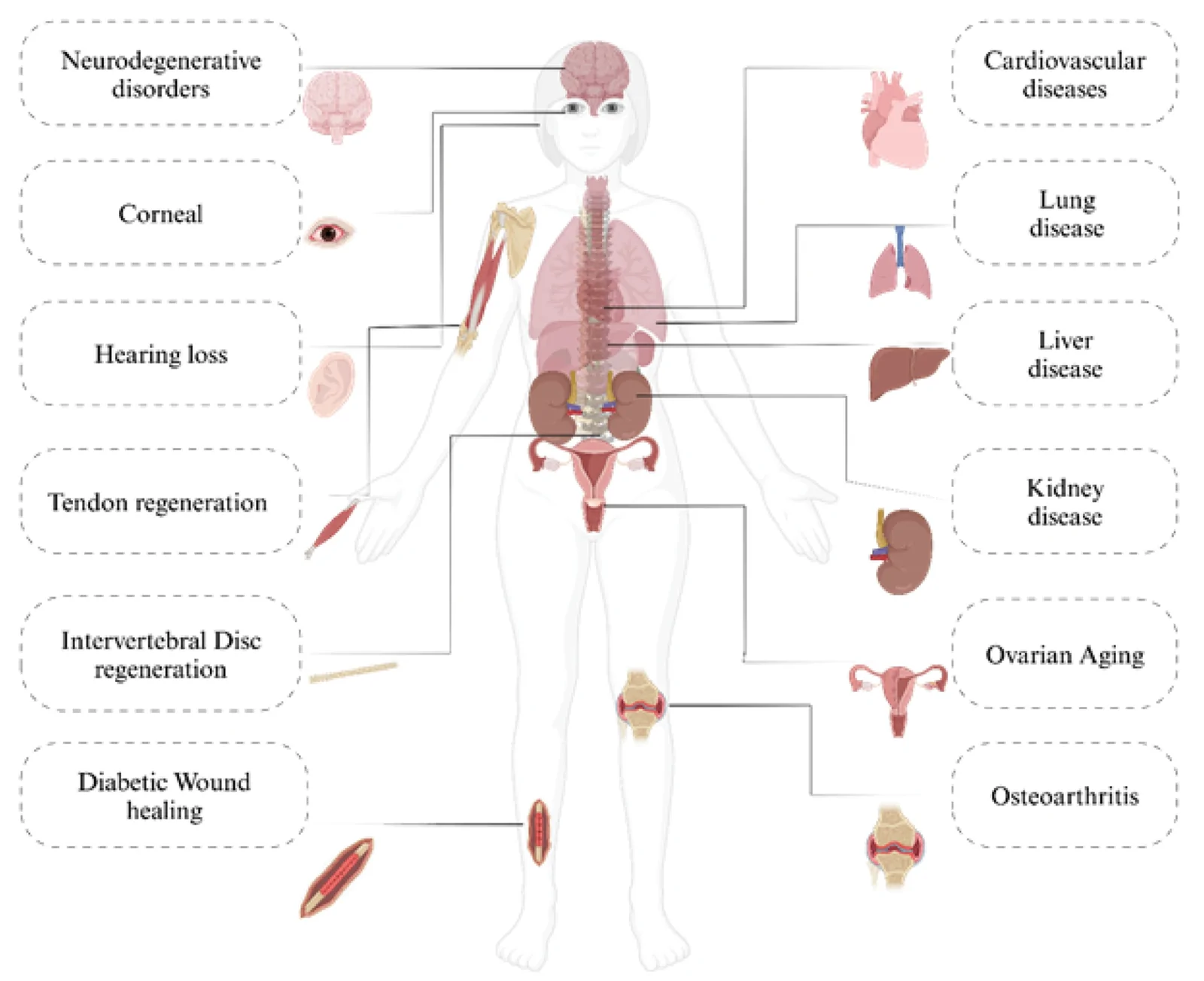
**Ferroptosis and Senescence in Human Diseases**. The figure illustrates the role of ferroptosis and cellular senescence in the pathogenesis of various human diseases. Ferroptosis and senescence both processes contribute significantly to this disease.

Collectively, these advances highlight the therapeutic promise of ferroptosis as a precision senolytics strategy. By targeting the senescence-ferroptosis nexus, innovative approaches ranging from nanoparticle delivery to combinatorial therapies can address diverse age-related diseases (Fig. 5). However, realizing this potential requires rigorous validation in disease-specific models and clinical trials to ensure efficacy and safety, positioning ferroptosis as a cornerstone of next-generation aging therapeutics.

## 6. Limitations and Opportunities for Ferroptosis in Clinical Application

The translation of ferroptosis-mediated SCs elimination from preclinical promise to clinical reality is fraught with challenges, yet it offers transformative opportunities. The complex interplay between ferroptosis and cellular senescence, compounded by the lack of universal biomarkers, hinders the design of robust preclinical and clinical studies [115, 116]. SC heterogeneity driven by cell type, tissue origin, senescence induction, and health status leads to variable responses to ferroptosis inducers like RSL3, which non-specifically inhibit glutathione peroxidase 4 (GPX4), risking toxicity to healthy cells [83, 84]. SASP further complicates efficacy by modulating drug responses and fostering resistance through adaptive signaling pathways [117]. Additionally, the reliance on in vitro and in vivo models that poorly recapitulate aged tissue pathophysiology limits translational insights. Systemic iron release from ferroptosis induction raises long-term safety concerns, particularly for chronic conditions requiring repeated dosing.

These limitations are offset by significant opportunities enabled by recent advancements. The iron-rich microenvironment and lipid dysregulation of SCs render them uniquely susceptible to ferroptosis, providing a therapeutic window for targeted senolytics [118-120]. High-throughput screening, coupled with multi-omics and single-cell analyses (DNA barcoding), facilitates the development of selective ferroptosis inducers targeting SC-specific pathways like ferritinophagy or ACSL4-mediated lipid peroxidation [121, 122]. Advanced drug delivery systems, such as nanoparticles and proteolysis-targeting chimeras (PROTACs), enhance specificity and bioavailability, mitigating off-target effects [123]. Repurposing existing ferroptosis inducers or conjugating them with senescence-specific markers further optimizes efficacy [124, 125].

Combinatorial strategies offer additional promise. Integrating ferroptosis inducers with cell cycle inhibitors, SASP suppressors, or immune modulators can overcome resistance and enhance SC clearance [89]. The ‘two-punch’ approach—inducing senescence followed by ferroptosis-mediated elimination shows potential in oncology, with applications in fibrosis and neurodegeneration [126, 127]. Artificial intelligence and deep learning accelerate drug discovery by predicting SC-specific responses and toxicity profiles, streamlining clinical development [128, 129]. Moreover, ferroptosis-based senolytics could enhance organ transplantation by clearing SCs from age donor tissues, addressing global organ shortages [114].

To capitalize on these opportunities, rigorous investigation is needed. Developing advanced models (e.g., organoids, aged mice) that capture SC heterogeneity, identifying biomarkers (4-HNE and MDA) for monitoring ferroptosis, and profiling long-term safety of iron-mediated therapies are critical steps. By leveraging interdisciplinary innovations, ferroptosis can overcome its current limitations, paving the way for precision senolytics that transform the treatment of age-related diseases.

## 7. Conclusions and Future Directions

The advent of ferroptosis as a senolytic strategy marks a paradigm shift in combating age-related diseases by exploiting the unique vulnerabilities of SCs. Unlike traditional senolytics, which rely on apoptosis and often face challenges such as poor specificity and resistance due to upregulated anti-apoptotic pathways, ferroptosis leverages iron-dependent lipid peroxidation to induce non-apoptotic cell death. This approach capitalizes on the elevated iron levels and disrupted lipid homeostasis inherent to SCs, offering a precise and effective means to clear these pathogenic cells. By synthesizing recent evidence, this review underscores ferroptosis’s potential to not only eliminate SCs but also delay senescence, paving the way for transformative therapies across a spectrum of age-related pathologies, including fibrosis, cancer, neurodegeneration, and beyond.

The therapeutic promise of ferroptosis extends beyond SC clearance. Emerging studies suggest that ferroptosis-mediated removal of SCs from aged donor organs could enhance organ viability, addressing the critical shortage of transplantable organs. Moreover, combining ferroptosis inducers (e.g., erastin derivatives, RSL3) with existing senolytics, proteostasis modulators, or immunotherapies could amplify efficacy, targeting resistant SC populations and mitigating the SASP. These synergistic strategies hold immense potential to reshape the treatment landscape for aging-related disorders, offering hope for improved healthspan and quality of life.

Recent advances in artificial intelligence (AI) and deep learning models have revolutionized the design and development of small molecules and proteins [130], presenting unprecedented opportunities for creating selective senolytics and ferroptosis inducers or inhibitors. AI-driven platforms, such as generative adversarial networks and reinforcement learning [131, 132], enable the rapid identification of novel compounds targeting SC-specific pathways, such as ferritinophagy, ACSL4-mediated lipid peroxidation, or GPX4 regulation[133, 134]. These models predict molecular interactions, optimize pharmacokinetic properties, and minimize off-target effects, accelerating the discovery of ferroptosis modulators tailored to SCs’ iron-rich microenvironment. Similarly, deep learning-guided protein design facilitates the engineering of biologics, such as PROTACs or antibodies, to enhance ferroptosis specificity in aging tissues[135, 136]. By integrating multi-omics data (e.g., transcriptomics, proteomics), AI can map SC heterogeneity and predict tissue-specific responses, paving the way for personalized ferroptosis-based therapies [128, 137].

Despite its promise, ferroptosis-based senolytics face significant hurdles that must be addressed to achieve clinical success. Key challenges include the development of selective ferroptosis inducers to minimize off-target effects on healthy cells, the optimization of safe and efficient drug delivery systems such as nanoparticle-based platforms[138], and the establishment of robust in vitro and in vivo models to capture the heterogeneity of SCs across tissues. Variability in drug resistance among SC populations, SASP-driven adaptive signaling, and the potential for systemic iron overload further complicate translation. To overcome these barriers, future research should prioritize the following directions:

1. Development of Selective Inducers: Leverage high-throughput screening, CRISPR-based approaches, and AI-driven drug design to identify ferroptosis inducers that target SC-specific vulnerabilities, such as ferritinophagy or ACSL4-mediated PUFA synthesis, while sparing healthy cells[130]. Recent advances in targeted drug design and deep learning models provide a blueprint for achieving this precision[139-141].
2. Elucidation of Dual Roles: Investigate the dual capacity of ferroptosis to eliminate SCs and delay senescence onset. Single-cell RNA sequencing, proteomic studies, and AI-integrated multi-omics analyses can uncover how ferroptosis modulates SASP and tissue microenvironments, informing tailored therapeutic strategies.
3. Combination Therapies: Explore synergistic regimens combining ferroptosis inducers with senolytics (navitoclax), SASP inhibitors, or immunotherapies to enhance SC clearance and mitigate resistance [142-144]. Preclinical studies in disease-specific models (e.g., Alzheimer’s, pulmonary fibrosis) are critical to validate these approaches.
4. Robust Model Systems: Develop advanced in vitro (e.g., organoids) and in vivo (e.g., aged mouse models) platforms that recapitulate SC heterogeneity and tissue-specific responses to ferroptosis[142, 145]. These models, coupled with AI-driven predictive modeling, will enable rigorous evaluation of efficacy and safety[146, 147].
5. Clinical Translation and Biomarkers: Establish biomarkers (4-HNE and MDA) to monitor ferroptosis efficacy and safety in clinical trials. Early-phase trials testing AI-designed ferroptosis inducers in aging-related diseases, inspired by ongoing cancer studies [148] should prioritize dose optimization and toxicity profiling.
6. Ethical and Accessibility Considerations: Address ethical challenges, such as ensuring equitable access to ferroptosis-based therapies and evaluating long-term effects in aging populations [103, 149]. Collaborative efforts between academia, industry, and policymakers are essential to navigate these complexities.

In conclusion, ferroptosis represents a groundbreaking approach to SCs elimination, offering a compelling alternative to traditional senolytics by targeting the iron-rich, lipid-altered microenvironment of SCs. The integration of AI and deep learning in designing small molecules and proteins further enhances the potential for developing selective, effective ferroptosis modulators, revolutionizing senolytic therapy. This review synthesizes the molecular underpinnings and therapeutic potential of ferroptosis, highlighting its capacity to address the root causes of age-related diseases. By advocating for rigorous preclinical and clinical investigations, leveraging interdisciplinary innovations, and harnessing AI-driven technologies, I envision a future where ferroptosis-based therapies not only clear pathological SCs but also rejuvenate tissues, enhance organ transplantation, and extend healthy longevity. The path forward demands collaborative efforts, innovative technologies, and a commitment to overcoming translational challenges. As I stand at the cusp of this therapeutic revolution, ferroptosis, empowered by AI, holds the key to unlocking new frontiers in aging research and precision medicine
